# Establishment of prognostic signature based on neutrophil extracellular traps-related genes in acute myeloid leukemia: a bioinformatics analysis

**DOI:** 10.3389/fimmu.2025.1580750

**Published:** 2025-10-24

**Authors:** Zhenglei Shen, Jingying Zhu, Ni Luo, Lei Feng, Heng Yue, Liying Song, Kunmei Liu, Huaxian Li, Honghua Cao, Yeying Zhou, Yasar Mehmood Yousafzai, Zia Asad, Youyu Qiu, Shiwen Zhang

**Affiliations:** ^1^ Department of Hematology, the Third Affiliated Hospital of Kunming Medical University, Yunnan Cancer Hospital, Peking University Cancer Hospital Yunnan, Kunming, China; ^2^ Department of Head and Neck Thyroid Surgery Department, The Third Affiliated Hospital of Kunming Medical University, Yunnan Cancer Hospital, Peking University Cancer Hospital Yunnan, Kunming, China; ^3^ Institute of Pathology and Diagnostic Medicine Khyber Medical University, Peshawar, Pakistan; ^4^ Molecular Biologist Public Health Reference Laboratory, Peshawar, Pakistan; ^5^ Radiology, Sun Yat-Sen Memorial Hospital, Sun Yat-Sen University, Guangzhou, China

**Keywords:** acute myeloid leukemia, neutrophil extracellular trap, prognostic signature, risk subgroups, tumor microenvironment

## Abstract

**Background:**

Acute myeloid leukemia (AML) is a hematological malignancy with a high incidence of febrile neutropenia during the first two treatment cycles. This study aims to develop a gene signature related to neutrophil extracellular traps (NETs) to enhance understanding of AML mechanisms and identify potential prognostic biomarkers.

**Methods:**

A consistent cluster analysis was conducted on 151 AML patients from the TCGA dataset. A differential analysis was performed to identify the differentially expressed genes (DEGs) specific to different subtypes and the training cohort (normal vs tumour). The NETs-related differentially expressed genes (NR-DEGs) were obtained through the overlapping of the two sets of differentially expressed genes. Univariate Cox and Least absolute shrinkage and selection operator (LASSO) regression analysis were employed to construct a NETs-related AML prognostic signature. Furthermore, an immune feature estimation and functional enrichment analysis was conducted between the two risk subgroups.

**Results:**

Two distinct AML subtypes were identified, exhibiting markedly disparate survival outcomes. A total of 1,700 and 1,941 DEGs were identified in the different subtypes and training cohort (normal vs. tumour), respectively. Thirteen NR-DEGs were identified. Subsequently, a NETs-related prognostic signature was constructed based on the 3 prognostic genes (*MPO*, *CCL3*, and *TLR8*). An independent prognostic analysis indicated that the risk score and age could be employed as independent prognostic factors. Our findings revealed the presence of five markedly differentially expressed immune cells between the two risk subgroups. Ultimately, it was determined that all three genes were associated with the ‘chemokine signalling pathway’.

**Conclusion:**

The prognostic signature comprised of *MPO*, *CCL3*, and *TLR8* based on NETs was established, which provided theoretical basis and reference value for the research of AML.

## 1 Introduction

Acute myeloid leukemia (AML) is a clonal hematopoietic stem cell disorder marked by the rapid proliferation of immature myeloid cells (1). The cornerstone of AML treatment has traditionally been chemotherapy; however, many patients experience drug resistance and high relapse rates, contributing to persistently poor overall survival outcomes (2). Recently, advancements in therapeutic strategies, including molecularly targeted therapies, specific antibodies, and immune checkpoint inhibitors, have aimed to address these resistance issues and enhance treatment efficacy (3). Furthermore, research has identified ALOX5AP as a promising prognostic marker in AML, indicating its potential role as a therapeutic target (4). Despite these advancements, the aggressive nature of AML and the scarcity of effective prognostic markers present significant challenges in improving patient survival rates. Thus, identifying new prognostic genes is crucial for monitoring patient outcomes and understanding the pathogenesis of AML.

The immune system’s role in AML has become a critical area of research, with immune dysregulation identified as a key factor in disease progression and treatment resistance (5). Neutrophils, the predominant immune cells in the bone marrow and peripheral blood, have a multifaceted role in cancer initiation, progression, and dissemination. In particular, neutrophil extracellular traps (NETs), which consist of decondensed chromatin enriched with antimicrobial proteins, have emerged as significant players in inflammation and cancer advancement (6). In the context of AML, NETs have been shown to modulate the immune response and may profoundly influence patient outcomes (7). However, the genes regulating NET formation and their prognostic implications in AML remain poorly understood. This knowledge gap has prompted our investigation into the potential of NETs-related genes as innovative prognostic biomarkers in AML (8).

In this study, we aimed to identify a NETs-related prognostic signature that could predict prognosis in AML patients By leveraging bioinformatics analyses on large-scale gene expression datasets, we sought to identify prognostic genes related to NETs in AML, establish a prognostic model and uncover the molecular underpinnings of these genes in AML and explore their potential as therapeutic targets. These findings contribute to offer a new perspective on the role of the immune system in AML prognosis.

## 2 Materials and methods

### 2.1 Data source

The TCGA database (https://www.cancer.gov/ccg/research/genome-sequencing/tcga) was utilized to obtain gene expression profiles from 151 blood samples of AML patients. Additionally, RNA sequencing data from 386 healthy peripheral blood samples were sourced from the GTEx database (https://commonfund.nih.gov/GTEx). The merged dataset from these sources was designated as the training cohort (batch effects were corrected for TCGA and GTEx data through the ComBat method). For validation of the risk model, gene expression data from the GSE71014 dataset (platform: GPL10558) were collected from the GEO database (https://www.ncbi.nlm.nih.gov/geo/) , which included 104 bone marrow samples. A total of 136 neutrophil extracellular trap (NET)-related genes (NRGs) were derived from the existing literature (9).

### 2.2 Consensus clustering of the NRGs model

The ‘ConsensusClusterPlus’ package was employed to conduct consensus cluster analysis on AML samples (N=151), utilizing the expression levels of all genes available in the TCGA database. Additionally, the ‘survminer’ package (10) was utilized to analyze Kaplan-Meier (KM) survival curves, focusing on overall survival (OS) across different subtypes (p <0.05). Subsequently, the ‘estimate’ package was implemented to compare Immune, Stromal, and ESTIMATE scores among the various subtypes via the Wilcoxon rank-sum test (p <0.05) (11).

### 2.3 Differential analysis

The ‘DESeq2’ package (12) was utilized to identify differentially expressed genes (DEGs) across different subtypes and within the training cohort (normal vs. tumor). The criteria for significance were set as |log_2_FC| > 1 and adjusted p < 0.05. Both volcano plots and heat maps were generated to illustrate the identified DEGs.

### 2.4 Gene functional enrichment analysis

NETs-related differential genes (NR-DEGs) were derived by intersecting NRGs and DEGs from different subtypes and the training cohort. Gene Ontology (GO) and Kyoto Encyclopedia of Genes and Genomes (KEGG) enrichment analyses of the NR-DEGs were performed using the ‘clusterProfiler’ package (13). Adjusted p < 0.05 were considered statistically significant.

### 2.5 Risk score-based subgroup analysis of NR-reated AML patients

Univariate Cox regression analysis was performed on NRG-DEGs to obtain candidate prognostic genes (p < 0.05, Hazard Ratio (HR) ≠ 1). Least Absolute Shrinkage and Selection Operator (LASSO) regression analysis was conducted based on candidate prognostic genes. Ten-fold cross-validation was adopted to screen prognostic genes based on the optimal lambda value. After screening out the prognostic genes, the risk scores of AML patients with available survival data were calculated based on the LASSO coefficient of each prognostic gene and the expression level of the genes. Then, AML patients with available survival data were categorized into two risk subgroups (low-risk vs. high-risk) based on the median risk score calculated per sample to construct a risk model. The prognostic reliability of the risk score and model was assessed by K-M and receiver operating characteristic (ROC) curve (1-, 3-, 5-years), respectively. Additionally, after cross-platform normalization of the GSE71014 dataset using the normalizeBetweenArrays function in the ‘limma’ package, external validation of the risk model was performed.

### 2.6 Clinical nomogram model

Univariate Cox regression analysis was performed for Risk score, gender, age, FAB category, and cytogenetics risk. Clinical factors with p < 0.05 and HR ≠ 1 were selected for proportional hazards (PH) test. Subsequently, multivariate Cox regression analysis was conducted on the clinical factors that passed the PH test. Clinical factors with p < 0.05 and HR ≠ 0 were selected as independent prognostic factors. The nomogram containing independent factors was drawn to predict 1-, 3-, and 5-year survival probability of AML patients. Evaluation of the predictive effect was done by the calibration curve. If the slope of the calibration curve was close to 1, it indicated that the prediction performance of the nomogram was good.

### 2.7 Gene set enrichment analysis

To explore the potential biological pathways involving prognostic genes, GSEA was performed in the training set. First, the correlation between each single prognostic gene and all genes in the training set was calculated, and then all genes were ranked from largest to smallest according to the correlation coefficients. Finally, GSEA was performed using the ‘clusterProfiler’ package (14). Adjusted p < 0.05 were deemed statistically significant.

### 2.8 Immune feature estimation and chemotherapy analysis

The relative abundance of 22 infiltrating immune cell types across different risk groups was assessed using the CIBERSORT algorithm (15). The Wilcoxon rank-sum test was used to compare the infiltration differences of 22 types of immune cells between the high-risk and low-risk groups, and the Benjamini Hochberg (BH) method was employed to correct the p-values (adjusted p < 0.05). Additionally, the expression levels of 20 immune checkpoint molecules were compared between the high- and low-risk groups (p < 0.05) (16). Standardized gene expression data were submitted to the tumor immune dysfunction and exclusion (TIDE) official website to obtain the TIDE score for AML, and the correlation between TIDE and risk score was analyzed.

### 2.9 Potential drug prediction analysis

Using the DGIdb database, we identified potential targeted therapies to explore prospective treatment options for the prognostic genes associated with AML.

### 2.10 Statistical analysis

In this study, the Wilcoxon rank-sum test was used to compare differences between the two groups, and the Log-rank test was employed to evaluate survival differences among different groups. A P-value less than 0.05 was regarded as statistically significant.

## 3 Results

### 3.1 The NRGs associated with prognosis in AML

**Figure 1:**
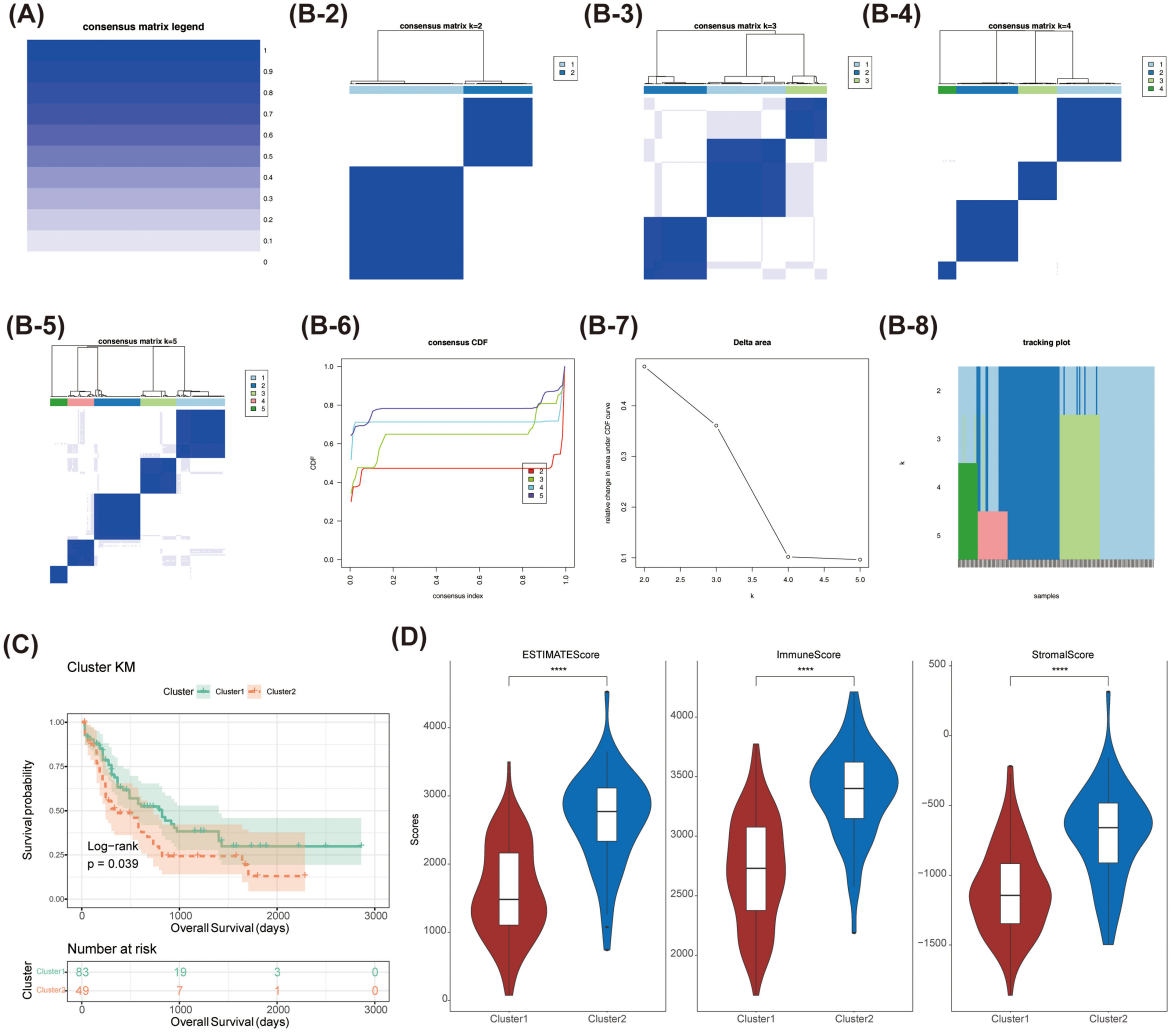
The consensus clustering analysis of myeloid leukaemia (AML) was conducted. **(A-B-2-8)**Comparison of clinical characteristics of patients with different subtypes of acute myeloid leukaemia (AML) **(C)** Survival analysis of patients with different subtypes of acute myeloid leukaemia (AML). The survival probability of Cluster 1 was higher than that of Cluster 2 (Log-rank p = 0.039). **(D)** Comparison of immune score and stromal score in patients with different subtypes of acute myeloid leukaemia (AML). **** stands for p < 0.0001.

Consensus clustering analysis identified two distinct subtypes among 151 AML patients: Cluster 1 (comprising 94 samples) and Cluster 2 (comprising 57 samples), based on the expression levels of 58,387 genes (**Figures 1A, B**). Uniform Manifold Approximation and Projection (UMAP) provided clear differentiation between these 2 clusters (**Supplementary Figure 1**). Kaplan-Meier (K-M) survival analysis indicated a significant difference in survival between Cluster 1 and Cluster 2, with patients in Cluster 2 exhibiting poorer OS probabilities (**Figure 1C**). Additionally, the distribution of various clinicopathological features was presented for each subtype (**Table 1**), revealing notable disparities in gender and age. Importantly, we observed that immune, stromal, and ESTIMATE scores were significantly elevated in Cluster 2 relative to Cluster 1 (**Figure 1D**).

**Table 1:** Comparison of clinical characteristics among different subtypes of acute myeloid leukemia (AML) patients.

Clinical features	Category	Total	Cluster1	Cluster2	pvalue
Gender	Female	68	45	23	0.3712
	Male	83	49	34	
Age (year)Mean (SD)		54.1 (± 16.1)	50.7 (± 15.9)	59.9 (± 14.7)	
	>=60	67	33	34	0.0005
	<60	84	61	23	
Vital	Alive	54	39	15	
	Dead	97	55	42	
WBC (10^9/L)	Mean (SD)	31.6 (± 37.3)	25.1 (± 42.2)	34.4 (± 39.8)	
	<4	30	26	4	0.0118
	>=4,<=10	23	12	11	
	>10	74	41	33	
	NA	24	15	9	
HB	Mean (SD)	9.57 (± 1.39)	9.71 (± 1.36)	9.33 (± 1.40)	0.1403
PB_blast (%)	Mean (SD)	38.9 (± 31.2)	43.7 (± 32.2)	31.0 (± 28.1)	0.0155
Platelet (10^9/L)	Mean (SD)	64.4 (± 53.1)	62.1 (± 49.3)	68.2 (± 59.2)	0.4958
OS (months)	Mean (SD)	18.83 (± 19.72)	21.44 (± 20.60)	14.94 (± 17.80)	0.0539
FAB	M0	15	12	3	
	M1	35	22	13	
	M2	38	31	7	
	M3	15	14	1	
	M4	29	8	21	
	M5	15	3	12	
	M6	2	2	0	
	M7	1	1	0	
	NA	1	1	0	

### 3.2 Identification of NR-DEGs in AML

**Figure 2:**
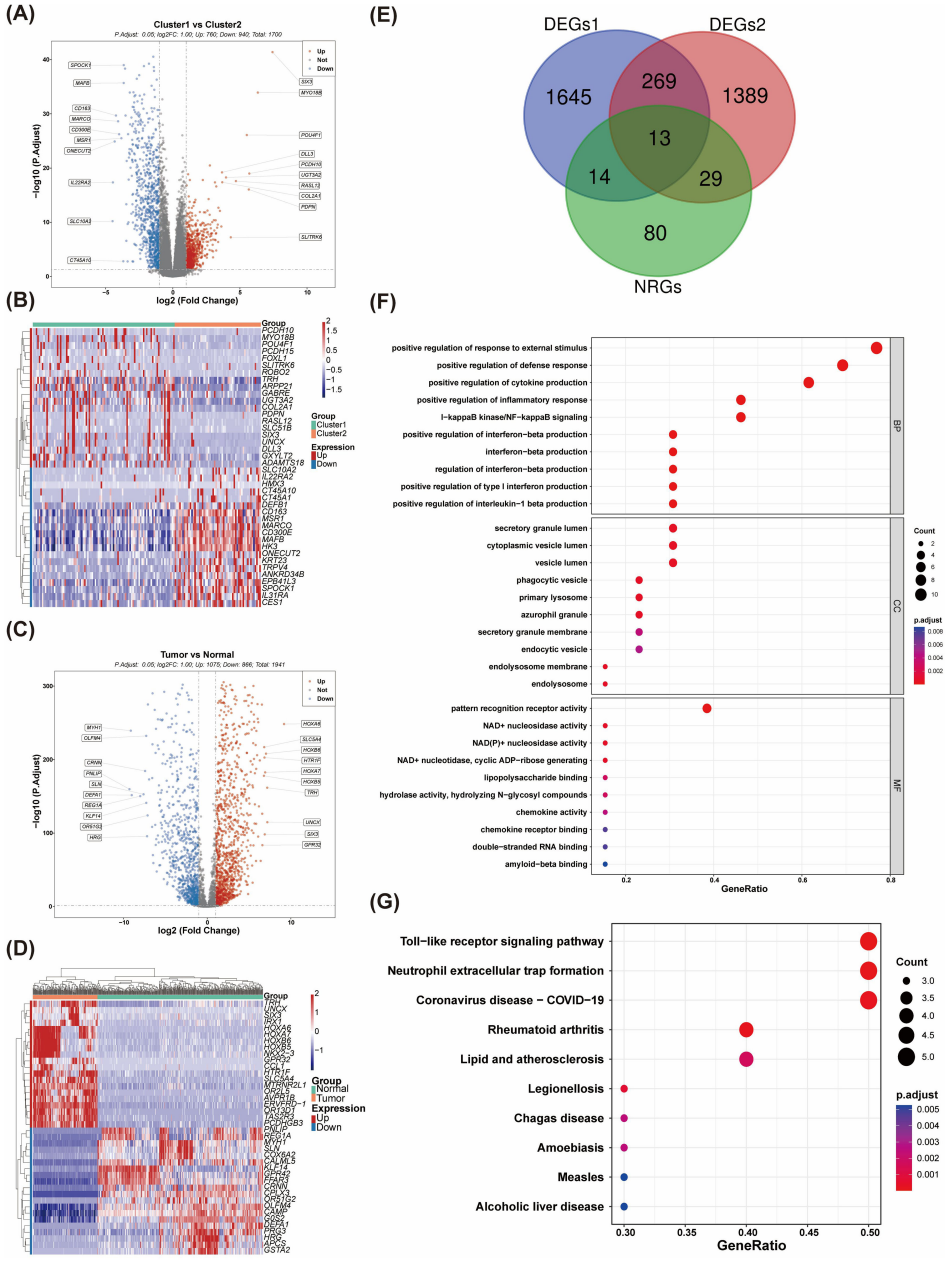
The identification and enrichment analysis of NR-DEGs. **(A)** The volcano plot of differentially expressed genes (DEGs) between Cluster 1 and Cluster 2. **(B)** The expression heatmap of DEGs between Cluster 1 and Cluster 2. **(C)** Volcano plots were generated to visualize the DEGs between AML and control samples. **(D)** Differential gene expression profiles in AML vs. normal tissues **(E)** A Venn diagram was constructed, revealing that a total of 13 NR-DEGs were identified. **(F, G)** Functional enrichment analyses were performed on the NR-DEGs through (GO) and Kyoto Encyclopedia of Genes and Genomes KEGG pathway analyses.

A total of 1,700 DEGs were identified between the two clusters, comprising 760 upregulated and 940 downregulated genes (**Figures 2A, B**). In a parallel analysis, 1,941 DEGs (1,075 upregulated and 866 downregulated) were detected between normal and tumor groups (**Figures 2C, D**). Ultimately, we identified 13 NR-DEGs (**Figure 2E**). Functional enrichment analysis was performed to further elucidate the roles of these NR-DEGs in AML. The top 10 items for each classification were illustrated in a bubble diagram (**Figure 2F**; **Supplementary Figures 2A, B**). Our analysis revealed that these genes were primarily associated with the ‘positive regulation of inflammatory response’ and ‘positive regulation of cytokine production.’ Furthermore, these genes were implicated in the ‘Toll-like receptor signaling pathway’ and the formation of ‘Neutrophil extracellular traps’ (**Figure 2G**; **Supplementary Figures 2C, D**).

### 3.3 NR-DEGs-based gene signature for AML outcome prediction

**Figure 3:**
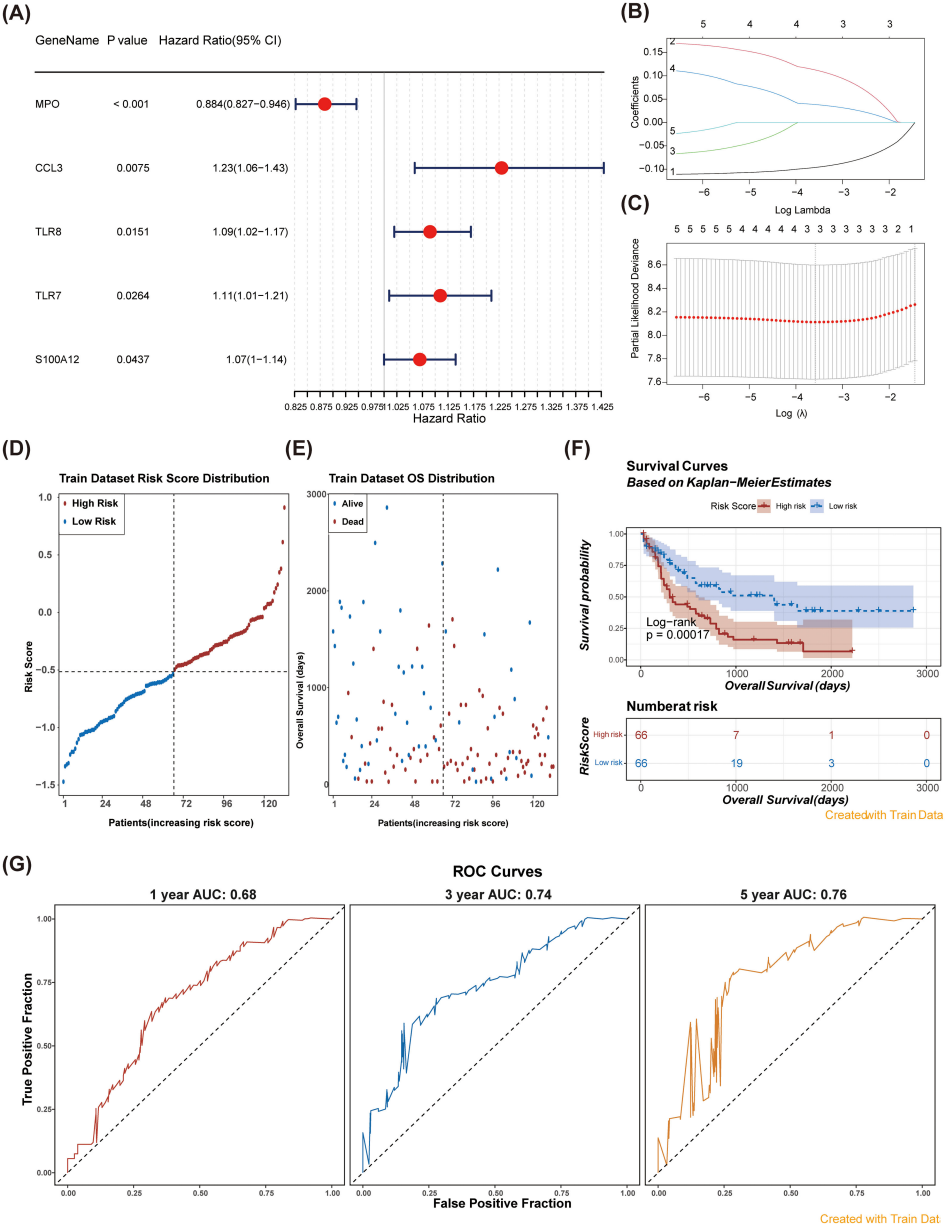
Identification of prognostic genes and construction of a risk model were performed. **(A)** Forest plots for univariate Cox regression analysis of NR-DEGs. Univariate Cox regression analysis identified five significant prognostic genes. **(B, C)** Least absolute shrinkage and selection operator (LASSO) analysis was performed on the five genes obtained from univariate Cox regression analysis. **(D)** Risk score distribution in patients: high vs. low risk groups over time. **(E)** Overall survival distribution of patients by risk score in the training dataset. **(F)** Kaplan-Meier survival curves for high and low risk patients based on risk scores. The survival probability of the low-risk group was higher than that of the high-risk group (Log-rank p = 0.00017). **(G)** ROC Curves for model performance at 1, 3, and 5 years using training data. The AUC values were all greater than 0.6.

Univariate regression analysis identified five significant prognostic genes (HR ≠ 1 & P < 0.05) within the training cohort (**Figure 3A**). To extract key prognostic indicators, we conducted LASSO regression on these five significant genes. Ultimately, three predictive genes were identified: *MPO*, *CCL3*, and *TLR8* (**Figures 3B, C**). These genes were used to establish a NETs-related prognostic signature for AML. Patients were classified into two risk groups (high-risk and low-risk) based on the gene signature (**Figures 3D, E**). Strikingly, significant survival differences were observed between the 2 risk groups, with high-risk patients demonstrating poorer survival outcomes (**Figure 3F**). To assess the reliability of the model, the area under the curve (AUC) values for predicting 1-, 3-, and 5-year survival rates of AML patients were 0.68, 0.74, and 0.76, respectively, in the training set, indicating the model’s predictive capability regarding the survival status of AML patients (**Figure 3G**).

**Figure 4:**
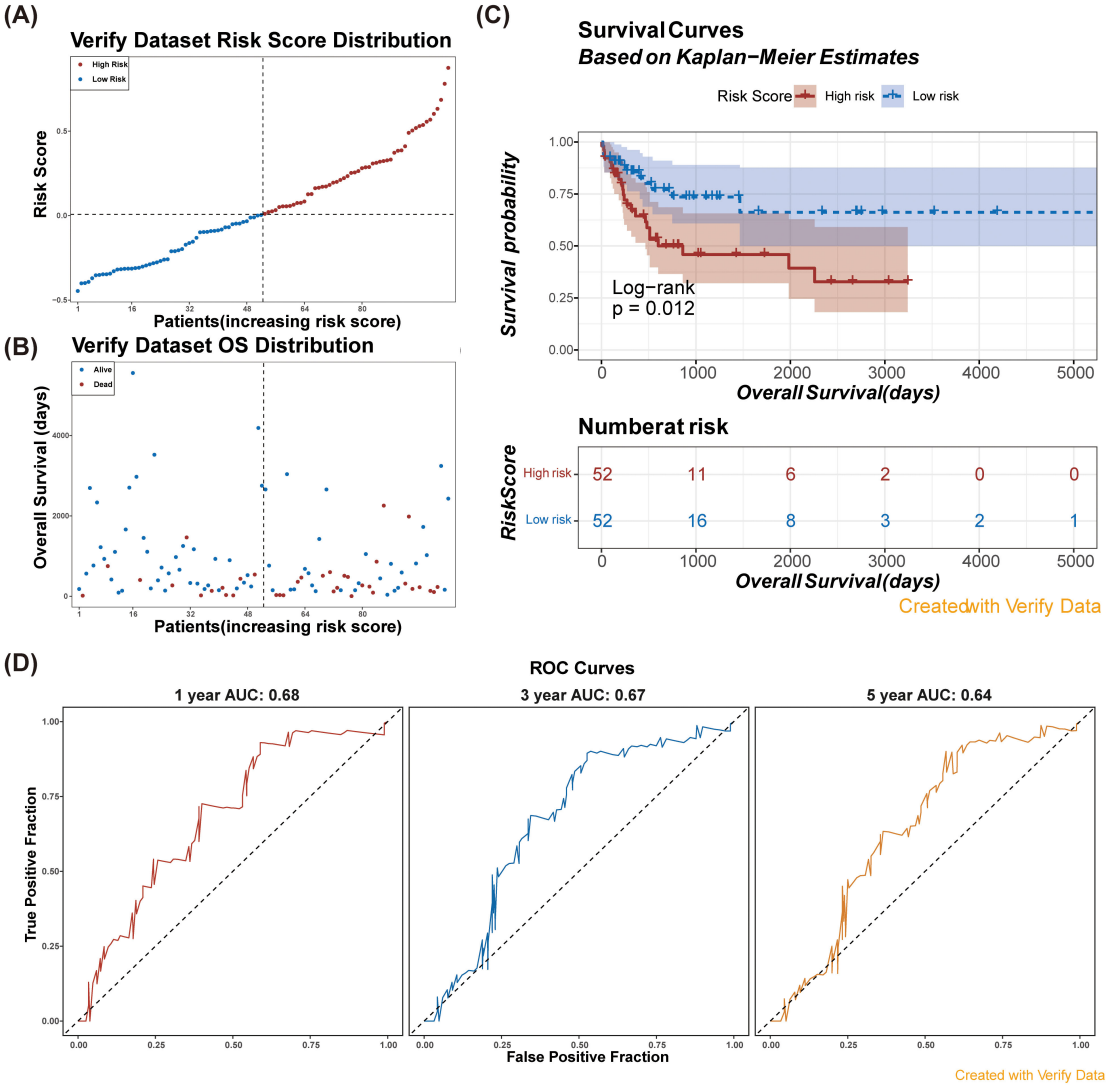
Verify the risk model in verification dataset (GSE71014). **(A)** Risk score distribution among patients in the verification dataset. **(B)** Overall survival distribution of patients in the verification dataset by risk score. **(C)** Kaplan-Meier survival curves for high and low risk patients based on risk scores in the verification dataset. Consistent with the results of the training set, the survival probability of the low-risk group was higher than that of the high-risk group.n **(D)** ROC curves for model performance at 1, 3, and 5 years using verification data.

Subsequently, we validated the risk model in an external dataset (GSE71014). Consistent with our training set findings (**Figures 4A, B**), patients in the high-risk group exhibited significantly poor overall survival (**Figure 4C**). The AUC values for the 1-, 3-, and 5-year survival predictions were consistently greater than 0.60 (**Figure 4D**), demonstrating that the NETs-related gene signature exhibited robust predictive performance in the validation cohort.

### 3.4 Independent prognostic analysis for AML patients

**Figure 5:**
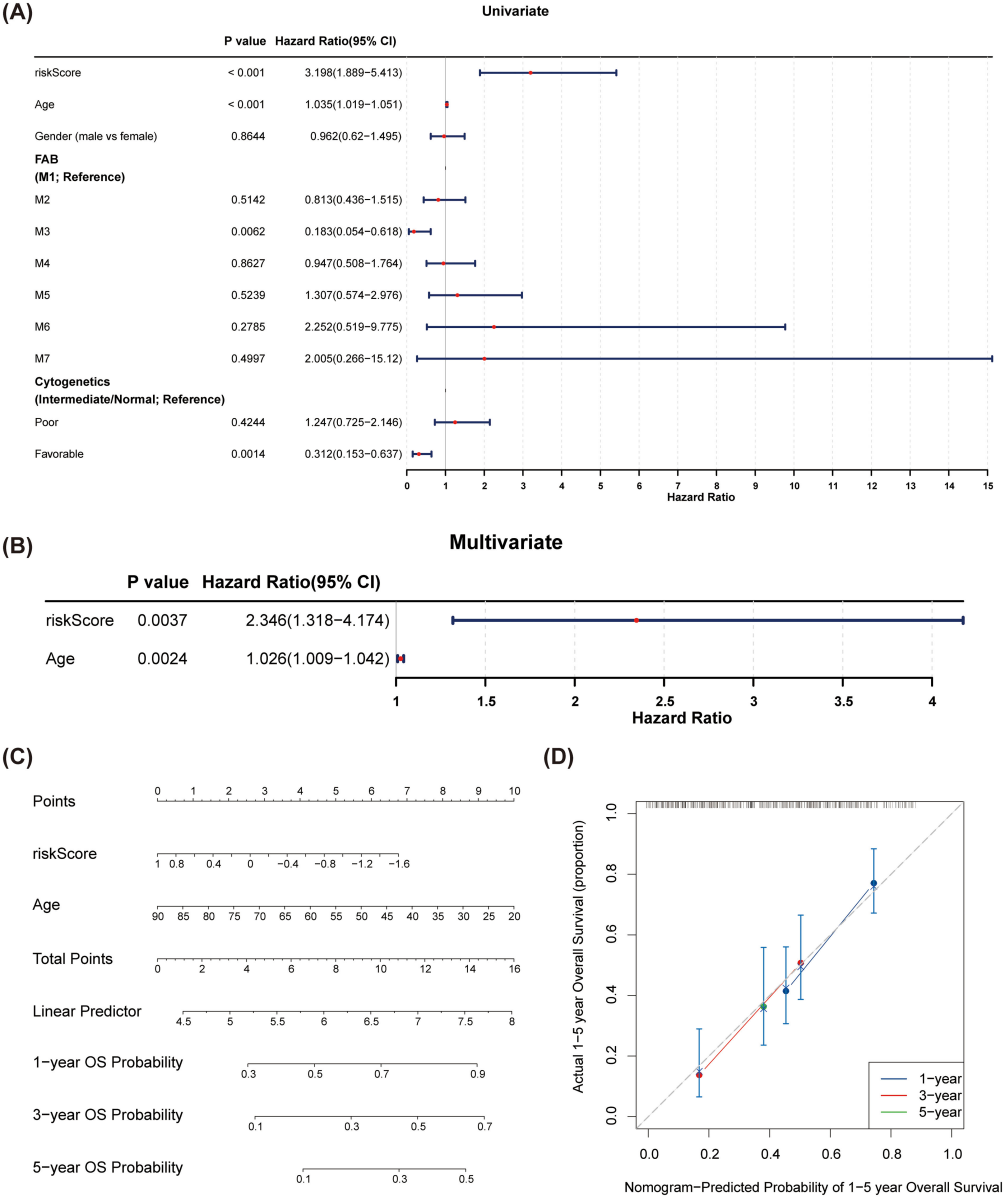
Construction and verification of the nomogram. **(A)** Forest plots of univariate Cox regression analyses for risk scores and clinical factors. Cytogenetics was divided into three categories: poor, intermediate, and favorable. The intermediate group was set as the reference group, and comparisons were made between poor vs. intermediate and favorable vs. intermediate. **(B)** Forest plots of multivariate Cox regression analyses for risk scores and clinical factors. Risk score and age were identified as independent prognostic factors. **(C)** A nomogram was constructed based on independent prognostic factors to predict the 1-, 3-, and 5-year overall survival probabilities of AML patients. **(D)** The calibration curves of the nomogram. The slopes of 1-, 3-, and 5-year survival were all close to 1, indicating high prediction accuracy of the nomogram.

Univariate Cox regression analyses indicated that risk score, cytogenetic risk, and age were significant prognostic factors (p < 0.05) (**Figure 5A**). The PH hypothesis test confirmed that risk score and age were appropriate for inclusion in the multivariate Cox regression model. These factors were validated as independent prognostic indicators for AML (**Figure 5B**). A nomogram depicting the predicted 1-, 3-, and 5-year survival rates was generated (**Figure 5C**), and the calibration curve demonstrated the accuracy and feasibility of the nomogram (**Figure 5D**).

### 3.5 The prognostic genes were related to chemokine relevant pathways

**Figure 6:**
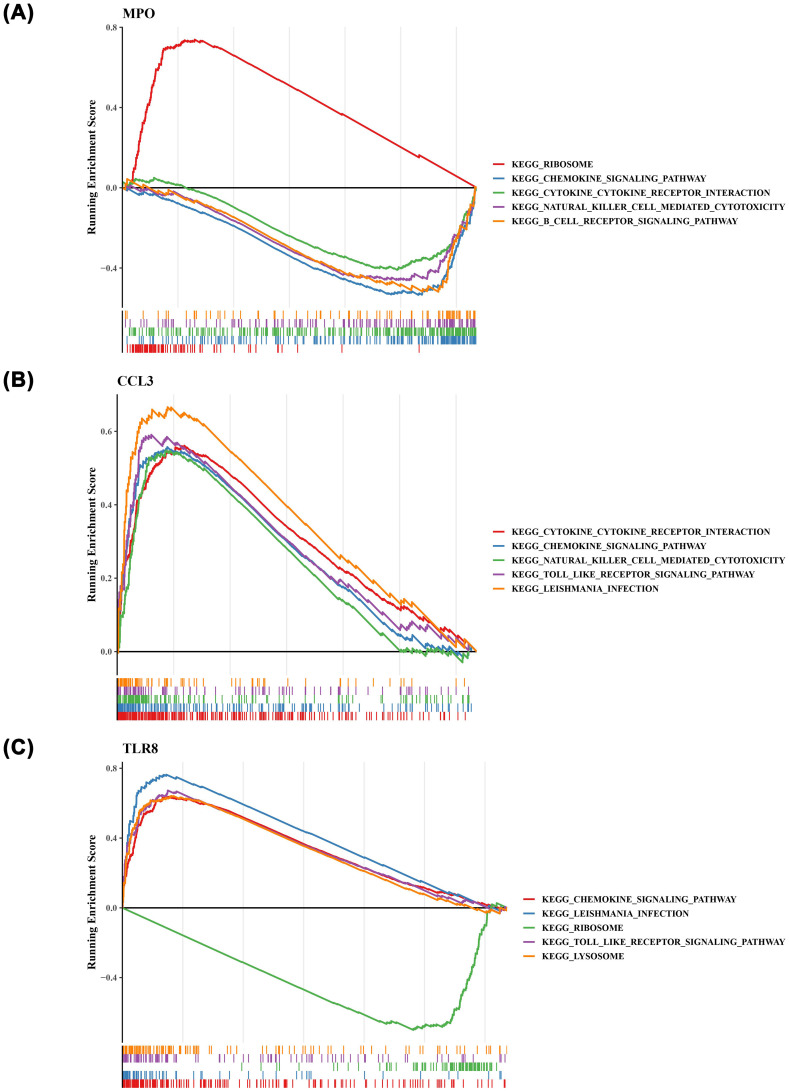
The gene set enrichment analysis (GSEA) result plots of the three prognostic genes. **(A)** MPO **(B)** CCL3 **(C)** TLR8. .

To explore the potential roles of the identified prognostic genes, we conducted GSEA on *MPO*, *CCL3*, and *TLR8*. Notably, we found that these three prognostic genes were significantly associated with the ‘chemokine signaling pathway’ (**Figure 6**). Furthermore, *MPO* (**Figure 6A**) and *CCL3* (**Figure 6B**) were linked to the ‘cytokine-cytokine receptor interaction’ pathway, while *TLR8* was enriched in the ‘Toll-like receptor signaling pathway’ (**Figure 6C**).

### 3.6 Immune infiltration analysis in risk subgroups

**Figure 7:**
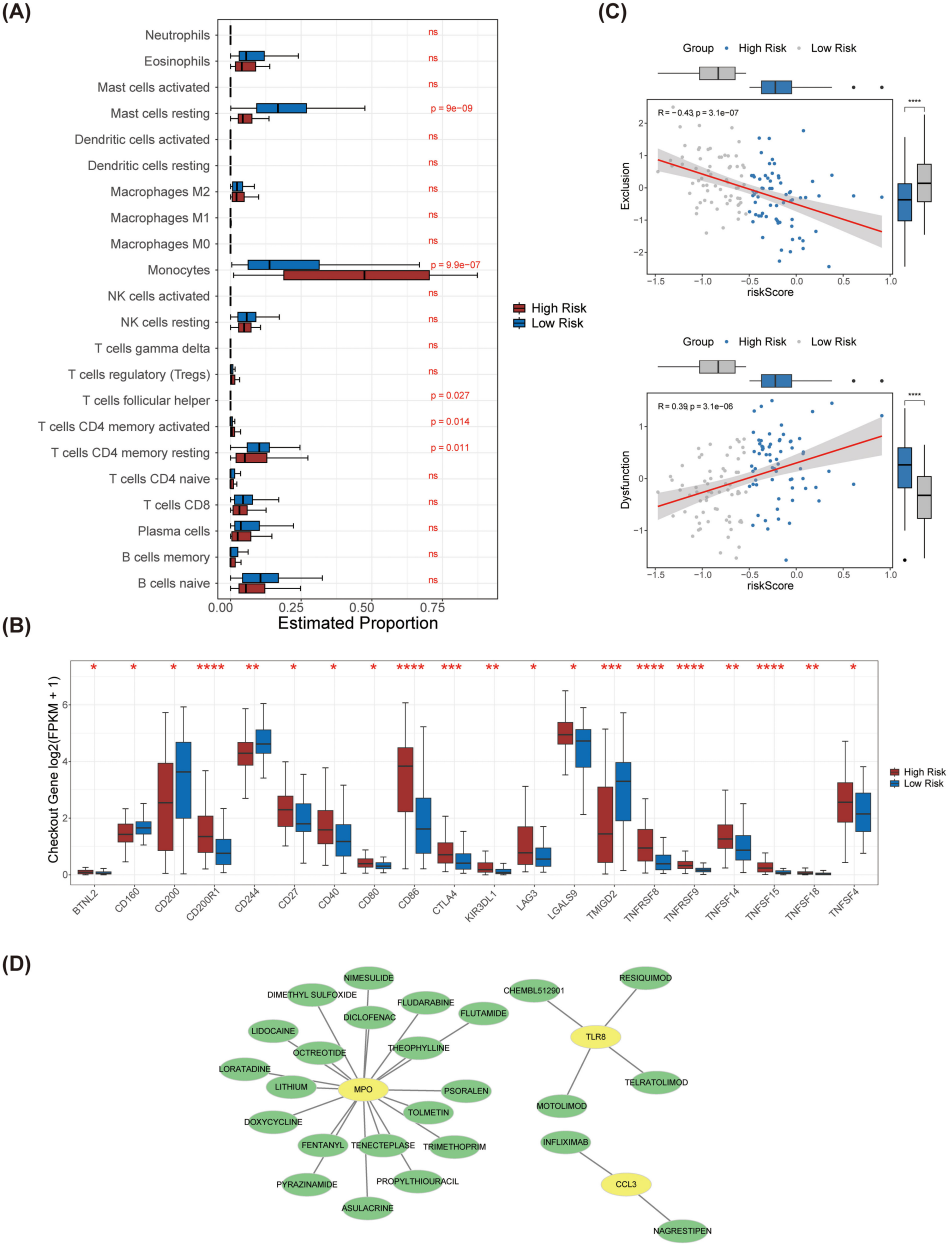
Immune infiltration analysis between high and low risk groups and drug prediction for prognostic genes. **(A)** The differences in the infiltration levels of 22 immune cells between the high and low-risk groups. **(B)** The differences in the expression of immune checkpoints between the high and low-risk groups. **(C)** Scatter plots of correlation analysis between exclusion, dysfunction and the risk score. Exclusion was significantly negatively correlated with the risk score, while dysfunction was significantly positively correlated with the risk score. **(D)** The prognostic gene-drug network diagram. Potential drugs were predicted for the three prognostic genes. **** represents p < 0.0001, *** p < 0.001, on behalf of ** p < 0.01, * on behalf of p < 0.05, ns on behalf of 0.05.

To investigate the immune microenvironment within AML, we analyzed the expression levels of 22 immune cell types across the 2 identified risk groups (**Supplementary Figure 3**). Our analysis revealed significant disparities in the abundances of five immune cell types, including resting mast cells, monocytes, follicular helper T cells, activated CD4 memory T cells, and resting CD4 memory T cells (**Figure 7A**). Additionally, all immune checkpoints exhibited differential expression between the high-risk and low-risk groups (**Figure 7B**). Importantly, we observed a strong positive correlation between the risk score and immune dysfunction, while the risk score showed a negative association with immune exclusion (**Figure 7C**).

### 3.7 Prognostic genes-drug network

With the use of the database, we discovered 25 potential medications that target *MPO*, *CCL3*, and *TLR8* (**Figure 7D**). The drugs that targeting *MPO* included tolmetin, asulacrine, and doxycycline etc. The drugs that targeting *TLR8* included CHEMBL512901, resiquimod, telratolimod, and motolimod. The drugs that targeting *CCL3* included infliximab and nagrestipen.

## 4 Discussion

This study presents novel insights into the prognostic gene expression patterns, biological functions, and potential clinical applications in AML through comprehensive bioinformatics analyses and extensive gene expression data. Our findings not only refine the molecular classification of AML but also reveal promising targets for future therapeutic strategies.

Utilizing consensus clustering analysis, we identified two distinct subtypes—Cluster 1 and Cluster 2—among a cohort of 151 AML patients based on the expression levels of 58,387 genes. This discovery represents a significant advancement in the molecular classification of AML, offering a novel approach to patient stratification driven by gene expression profiles. PCA further validated the clear segregation of these two clusters, corroborating existing literature on the inherent heterogeneity of AML. K-M survival analysis indicated a marked difference in survival outcomes between the two clusters, with patients in Cluster 2 demonstrating significantly poorer prognoses. These findings align with prior studies (3) linking specific AML subtypes to unfavorable outcomes. Notably, we observed significant variations in gender and age distributions between the two clusters, underscoring the potential impact of divergent biological characteristics and environmental factors on AML pathogenesis. Furthermore, Cluster 2 exhibited elevated immune, stromal, and ESTIMATE scores compared to Cluster 1, indicating a more active tumor microenvironment and a state of immune suppression. This observation echoes the work of Shaul and Fridlender (4), which highlights the role of tumor-associated neutrophils in shaping the tumor microenvironment and modulating the immune response.

In this study, we identified five significant genes through univariate regression analysis and further validated three key prognostic genes—Myeloperoxidase (*MPO*), Macrophage Inflammatory Protein-1α (*CCL3*), and Toll-Like Receptor 8 (*TLR8*)—using LASSO regression analysis. These genes were leveraged to construct a novel NETs-related prognostic signature for acute AML, marking a significant advancement in the molecular prognostic landscape of AML. Our prognostic model stratified AML patients into high-risk and low-risk groups, revealing significant survival disparities between these cohorts, with the high-risk group exhibiting markedly poorer outcomes. This finding is consistent with existing literature that underscores the relationship between specific gene expression patterns and AML prognosis (6). Our research not only delineates genes associated with AML prognosis but also formulates a robust risk model based on these markers. The model demonstrated commendable predictive performance, achieving AUC values of 0.68, 0.74, and 0.76 for 1-, 3-, and 5-year survival predictions, respectively. This is in line with the observations of Fu et al. (7), who classified AML through molecular subtypes that correlate with distinct prognoses. Our study enhances these findings by providing more precise prognostic insights through a dedicated gene signature. Validation of the risk model in the external dataset GSE71014 yielded results congruent with those from the training set, further affirming the robustness and reliability of our model.

*MPO*, a member of the myeloperoxidase protein family, is an enzyme predominantly expressed in neutrophils and is capable of generating reactive oxygen species (ROS) with potent oxidizing properties (8). Previous studies have shown that *MPO* and Fas/FasL expression can suppress CD4+ and CD8+ T cells, contributing to tumor progression (17).In the context of AML, *MPO* expression is closely associated with chemotherapy sensitivity, given its influence on ROS production within AML cells, which can consequently affect their responsiveness to chemotherapeutic agents. The findings of our study advocate for *MPO* as a prognostic marker in AML and provide a theoretical foundation for novel therapeutic strategies targeting ROS pathways. This aligns with previous research that emphasizes *MPO*’s significant role in AML prognosis. By incorporating *MPO* into our prognostic model, we further substantiate its critical involvement in treatment responses in AML.

*CCL3*, also referred to as Macrophage Inflammatory Protein-1α (MIP-1α), encodes a chemokine that directs the migration of immune cells, particularly leukocytes, to sites of inflammation or infection (9). In the realm of AML, the role of *CCL3* is especially pertinent; it not only facilitates immune cell recruitment but may also influence disease progression and patient prognosis. Our study revealed that *CCL3* expression in AML correlated significantly with patient risk scores and alterations in immune cell infiltration levels. This is supported by previous investigations demonstrating *CCL3*’s multifaceted role in various cancers, particularly regarding its regulation of the immune microenvironment in AML. Moreover, our study elucidates the direct association between *CCL3* and AML prognosis, offering new insights for future therapeutic strategies aimed at this disease.

*TLR8* is a pattern recognition receptor that plays a pivotal role in recognizing microbial components and activating the host’s innate immune response (10, 11). In the context of cancer, *TLR8* is implicated in the activation of antitumor immune responses. As a member of the Toll-like receptors (TLRs) family, *TLR8* interacts with damage-associated molecular patterns (DAMPs), modulating antigen presentation and cytokine release, thereby directly influencing T-cell activation and the clearance of tumor cells (18). Our study indicates that *TLR8* may significantly influence the immune microenvironment of AML, potentially impacting disease progression and treatment strategies. This finding aligns with previous research demonstrating *TLR8*’s critical role in the immune response to various cancers. By highlighting the association between *TLR8* and AML prognosis, our study offers a new theoretical foundation for the development of immunotherapies designed to leverage the innate immune system’s capabilities.

Univariate Cox regression analysis in this study identified risk score, cytogenetic risk, and age as significant prognostic factors in AML. These findings are consistent with the literature that emphasizes the importance of age and cytogenetic features in AML prognosis (12). For example, Nair et al. (13) noted in their review of AML treatment strategies that age is a key determinant of prognosis, often with older patients experiencing poorer outcomes. Furthermore, cytogenetic risk categories are a standard component of AML prognostic classification, closely correlated with treatment response and survival rates. Our study not only reaffirms the significance of these traditional prognostic factors but also establishes risk score and age as independent prognostic indicators in AML through PH hypothesis testing. This aligns with the findings of Kremer et al. (14, 19, 20), who reported that the regulation of the CXCR4 signaling pathway influences apoptosis in AML cells, potentially impacting prognosis. By providing a comprehensive risk score, our study refines prognostic assessments, enabling more accurate identification of high-risk patient populations. The generated nomogram forecasts 1-year, 3-year, and 5-year survival rates, while the calibration curve substantiates the nomogram’s effectiveness. The development of this prognostic tool is crucial, as it empowers clinicians and patients to better understand treatment outcome probabilities, aiding in informed decision-making. This aligns with research by Waugh and Wilson (15, 21), which discussed the role of the IL-8 pathway in cancer prognosis and treatment response. Our study extends these insights by offering a risk score-based prognostic model, providing a novel perspective for individualized treatment approaches in AML.

The GSEA of *MPO*, *CCL3* and *TLR8* revealed that these 3 prognostic genes were closely associated with the “chemokine signaling pathway, we conducted GSEA on *MPO*, *CCL3*, and *TLR8*, revealing that these 3 prognostic genes are closely linked to the “chemokine signaling pathway.” This discovery holds significant importance in AML research, as it elucidates potential mechanisms through which these genes may contribute to AML progression. Specifically, *MPO* and *CCL3* are associated with “cytokine-cytokine receptor interaction,” while *TLR8* is enriched in the “Toll-like receptor signaling pathway.” These results are in accordance with existing studies that emphasize the roles of chemokines and cytokines in shaping the immunological microenvironment and influencing disease progression in AML (16, 22).

In this study, we examined the immune microenvironment of AML patients by contrasting high-risk and low-risk subgroups, focusing on the expression levels of 22 immune cell types. Our analysis revealed significant differences in the abundances of five immune cell populations: resting mast cells, monocytes, follicular helper T cells, activated CD4 memory T cells, and resting CD4 memory T cells. These findings underscore the complexity of the immune landscape in AML and highlight the potential significance of various immune cell types in AML prognosis. Furthermore, we noted variations in the expression of immune checkpoints between the high-risk and low-risk groups, suggesting possible associations with immune escape mechanisms. Our study not only identifies the differential expression of immune cells but also correlates these findings with the risk scores of AML patients. This is in line with the work of Anderson et al. (23), who reported the expression of C-C chemokine receptor 1 in hematopoietic neoplasms, which may facilitate immune cell recruitment and influence tumor microenvironment regulation. Our research refines these observations by elucidating the relationship between specific immune cell subsets and AML risk scores, offering a fresh perspective for understanding the immune microenvironment in AML. Such insights can inform future treatment strategies; for instance, therapies targeting specific immune cell populations might effectively modulate the immune response, thereby enhancing the prognosis for AML patients. Our findings are corroborated by the research of Smits et al. (24), who demonstrated that the Toll-like receptor 7/8 agonist resiquimod boosts the immunostimulatory capacity of human AML cells, likely correlating with the differences in immune checkpoint expression identified in our study.

Through univariate regression analysis, five significant genes were identified, and 3 key prognostic genes were further confirmed—*MPO*, *CCL3*, and *TLR8* —using LASSO regression analysis. These genes were utilized to construct a NETs-related prognostic signature for AML, an innovative finding in the molecular prognostic research of AML. This prognostic model categorized AML patients into high-risk and low-risk groups, revealing significant survival differences between the 2 with the high-risk group having poorer outcomes. This result is consistent with existing studies that emphasize the association between specific gene expression patterns and AML prognosis (6). Our study not only identified genes associated with AML prognosis but also developed a risk model based on these genes. The model demonstrated good predictive performance, with AUC values of 0.68, 0.74, and 0.76 for 1-, 3-, and 5-year survival predictions, respectively. This is in agreement with the findings of Fu et al. (7), who classified AML through molecular subtypes and found these subtypes to be associated with different prognoses. Our study further refined these findings by providing more precise prognostic information through a specific gene signature. Validation of the risk model in an external dataset, GSE71014, yielded results consistent with the training set, further confirming the robustness and reliability of our model.

The significance of this study lies in the identification of a novel gene signature associated with prognosis in AML, as well as insights into how these genes regulate the immune microenvironment and influence disease progression. This discovery provides new molecular targets for personalized treatment strategies in AML, thereby enhancing patient treatment responses and outcomes. However, our study has certain limitations, including its retrospective design and reliance on publicly available datasets, which may introduce biases that affect the generalizability of our findings. Therefore, future research should aim to incorporate prospective study designs and larger, more diverse patient cohorts to validate our prognostic models. Meanwhile, verification is carried out in a sample queue containing rich stroma. Additionally, experimental studies, including *in vitro* and *in vivo* assays, are necessary to confirm the roles of *MPO*, *CCL3*, and *TLR8* in NETs and immune regulation in AML (25, 26). Clinical trials should also be considered to evaluate the practical utility of our prognostic model in guiding treatment decisions and improving patient outcomes (27, 28).

## 5 Conclusion

We identified 3 prognostic genes: *MPO*, *CCL3*, and *TLR8*. The expression of MPO is closely associated with chemotherapy sensitivity, potentially influencing the response of leukemia cells to chemotherapeutic agents by regulating the production of ROS. *CCL3* expression is significantly correlated with patient risk scores and levels of immune cell infiltration, which may affect disease progression and patient prognosis by modulating immune cell infiltration in the tumor microenvironment, potentially playing a role in immune evasion and progression of AML. *TLR8* may impact the tumor microenvironment by activating immune responses, thereby influencing disease progression and treatment strategies. The expression of TLR8 is closely related to the regulation of the immune microenvironment, which may provide a theoretical basis for developing new immunotherapeutic strategies. This study provides a novel prognostic signature for AML, offering insights into the molecular mechanisms underlying the disease and potential avenues for targeted therapy. Future research should focus on validating these findings in larger and more diverse cohorts, as well as exploring the functional roles of the identified genes in the progression of AML and the response to treatment.

## Data Availability

The datasets presented in this study can be found in online repositories. The names of the repository/repositories and accession number(s) can be found in the article/**Supplementary Material**.

## Supplementary material

Supplementary Figure 1 UMAP analysis plots of different subtypes.

Supplementary Figure 2 The GO analysis results of NR-DEGs. **(A)** The GO enrichment string graph of DEGs between Cluster 1 and Cluster 2. (B) The GO enrichment string graph of DEGs between AML samples and control samples. (C) Analysis of toll-like receptor signaling pathway and neutrophil extracellular trap formation. (D) Gene expression analysis of toll-like receptor signaling pathway and neutrophil extracellular trap formation.

Supplementary Figure 3 The proportion of 22 levels of immune cell infiltration in the samples.
