# Supplementary figures and images for: Establishment of prognostic signature based on neutrophil extracellular traps-related genes in acute myeloid leukemia: a bioinformatics analysis
Source: Front Immunol. 2025 Oct 24;16:1580750. doi: 10.3389/fimmu.2025.1580750 (PMC12592046; doi:10.3389/fimmu.2025.1580750)

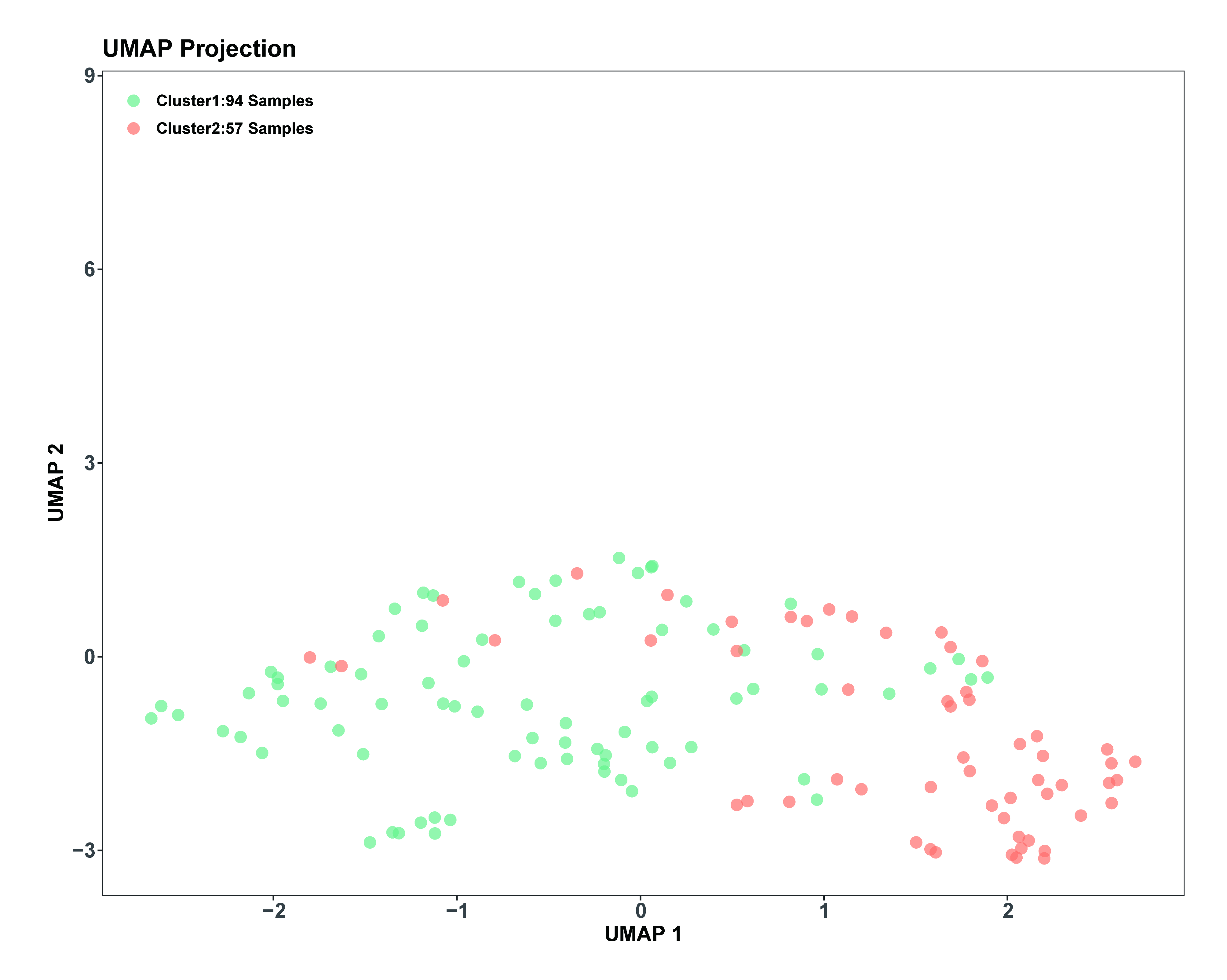

Supplement: Supplementary Figure 1 — UMAP analysis plots of different subtypes. [file Image1.tif]

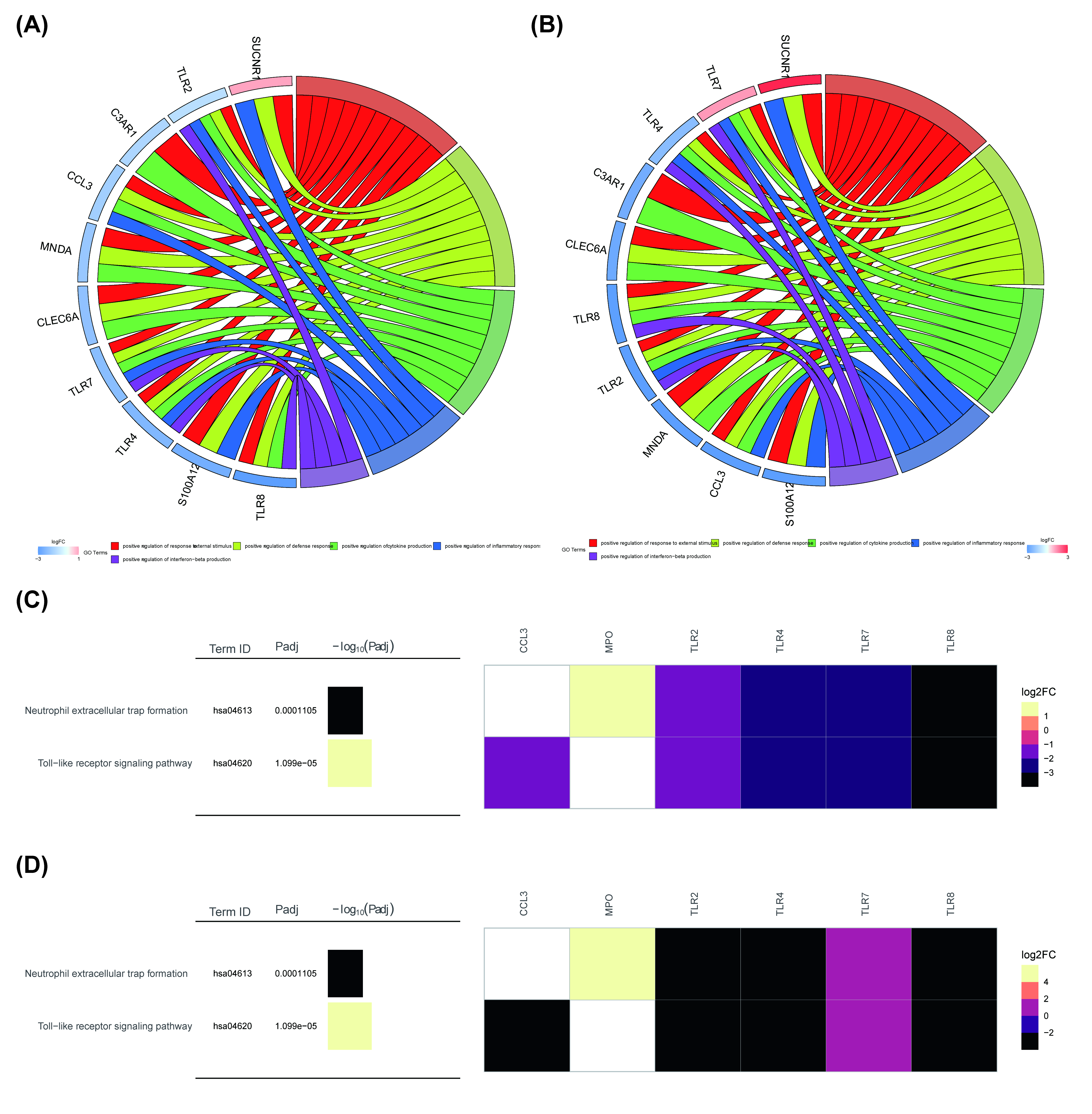

Supplement: Supplementary Figure 2 — The GO analysis results of NR-DEGs. (A) The GO enrichment string graph of DEGs between Cluster 1 and Cluster 2. (B) The GO enrichment string graph of DEGs between AML samples and control samples. (C) Analysis of toll-like receptor signaling pathway and neutrophil extracellular trap formation. (D) Gene expression analysis of toll-like receptor signaling pathway and neutrophil extracellular trap formation. [file Image2.tif]

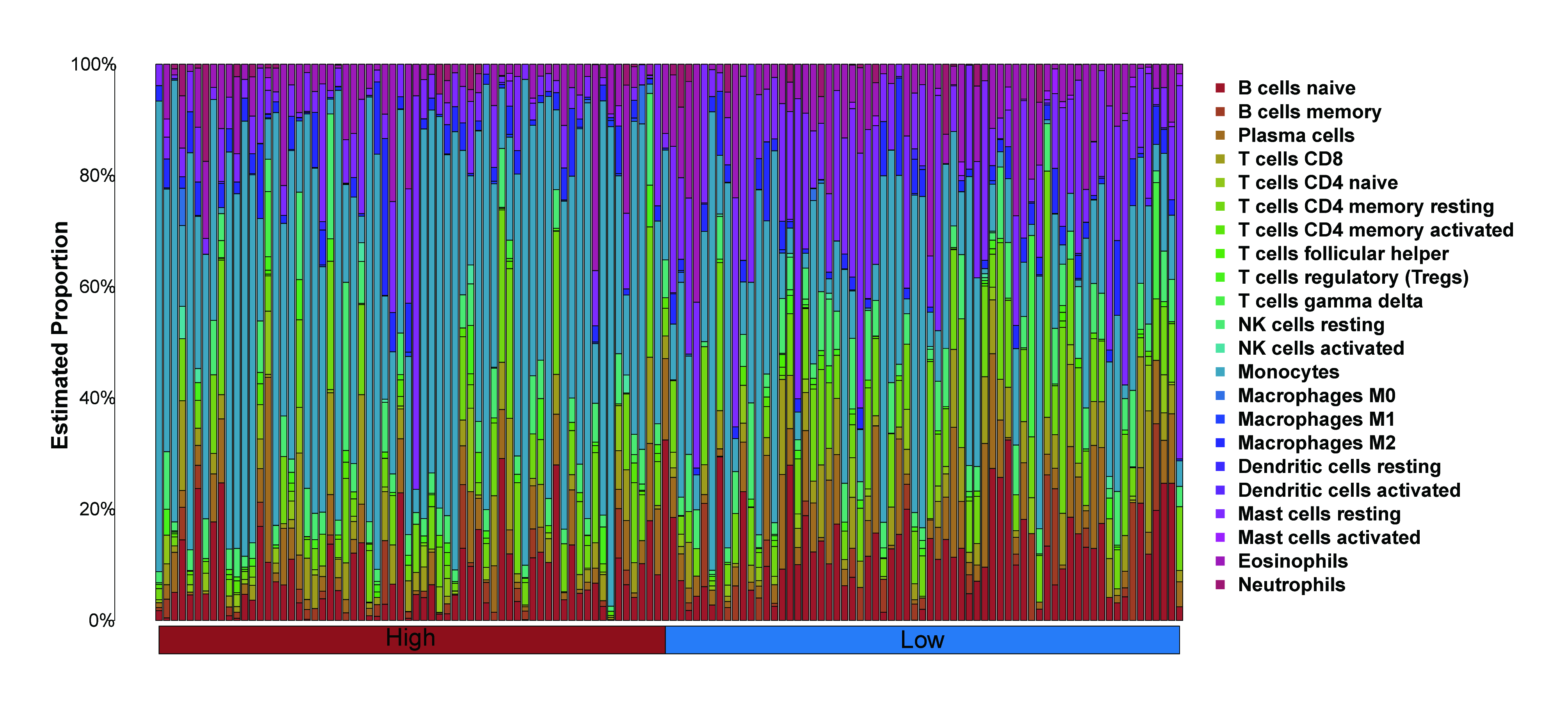

Supplement: Supplementary Figure 3 — The proportion of 22 levels of immune cell infiltration in the samples. [file Image3.tif]
